# Natural killer cell dysregulation in polycystic ovary syndrome: immunometabolic and reproductive implication

**DOI:** 10.3389/fimmu.2026.1781451

**Published:** 2026-04-15

**Authors:** Ningxiao Jiang, Jun Liu, Chanyu Li, Xiuju Wang, Yiwei Pang, Xianghui Zhang, Yixuan Ma, Shinan Zhang, Yingjiang Xu, Qingchun Li, Qinglei Sun, Peiyi Tang, Lei Han

**Affiliations:** 1 Department of Reproductive Medicine, Binzhou Medical University Hospital, Binzhou Medical University, Binzhou, Shandong, China; 2 Department of Gynecology, Hainan General Hospital, Hainan Affiliated Hospital of Hainan Medical University, Haikou, Hainan, China; 3 Department of Gynecology and Obstetrics, The First Affiliated Hospital of Chengdu Medical College, Chengdu, Sichuan, China; 4 Department of Interventional Vascular Surgery, Binzhou Medical University Hospital, Binzhou, Shandong, China; 5 Department of Gastrointestinal Surgery, Binzhou Medical University Hospital, Binzhou Medical University, Binzhou, Shandong, China; 6 Department of Clinical Nutrition, Chongqing University Jiangjin Hospital, Chongqing, China

**Keywords:** chronic inflammation, hyperandrogenism, immunometabolism, insulin resistance, natural killer cell, polycystic ovary syndrome, uterine NK cells

## Abstract

Polycystic ovary syndrome (PCOS), a complex endocrine and metabolic disorder, involves significant dysregulation of the immune system. Natural killer (NK) cells, as key components of innate immunity, demonstrate notable phenotypic and functional alterations in women with PCOS. These changes include not only an elevated proportion in peripheral blood but also dynamic shifts within the local microenvironments of the ovary and endometrium. The increased level of peripheral NK cells correlates with a chronic low-grade inflammatory state, potentially serving as a predictive marker in infertile PCOS patients. Within the endometrium, uterine NK (uNK) cells exhibit reduced numbers and impaired function, accompanied by dysregulation of cytokine networks such as IL-15 and IL-18, which disrupts the immune equilibrium essential for embryo implantation. Abnormal NK cell function further involves alterations in killer immunoglobulin-like receptor (KIR) repertoires and dysregulated secretion of angiogenic factors, thereby compromising endometrial receptivity and vascular remodeling. Hyperandrogenemia modulates the distribution and activity of NK cells in reproductive tissues by influencing their surface activation markers, while insulin resistance promotes the generation of myeloid-feature NK (myNK) cell subsets via the IL-6/Stat3 signaling pathway, collectively exacerbating metabolic inflammation and reproductive dysfunction. Deciphering the role of NK cells in the immunometabolic interplay of PCOS reveals their position as a critical link between. May represent a potential cutoff requiring validation in larger cohorts reproductive impairment and metabolic disturbances, opening new avenues for targeted immunomodulatory interventions. Collectively, NK cells appear to present an important immunometabolic link between reproductive dysfunction and metabolic disturbance in PCOS, highlight their potential relevance as therapeutic targets.

## 1 Introduction

Polycystic ovary syndrome (PCOS) represents a prevalent endocrine-metabolic disorder among women of reproductive age, characterized by hyperandrogenemia, ovulatory dysfunction, and polycystic ovarian morphology, often accompanied by metabolic disturbances such as insulin resistance (IR) (1). Emerging evidence underscores chronic low-grade inflammation as a central pathological link connecting reproductive impairments and metabolic complications in PCOS (2–4). Natural killer (NK) cells, a specialized subset of innate lymphoid cells, play crucial roles in maintaining female reproductive homeostasis by rapidly identifying and eliminating abnormal or infected cells (5). Uterine NK (uNK) cells, in particular, contribute to endometrial receptivity, vascular remodeling, and maternal-fetal immune tolerance (6, 7). However, the phenotypic alterations and functional deviations of NK cells in PCOS remain incompletely elucidated, especially regarding their interactions with androgens and insulin under PCOS conditions. This review systematically examines NK cell abnormalities in PCOS, dissects their interplay with hyperandrogenemia and IR, and explores their specific contributions to reproductive and metabolic dysregulation, thereby offering fresh insights into the immunopathological mechanisms of PCOS.

## 2 Evidence from preclinical and clinical studies

### 2.1 Animal evidence

Multiple animal models of polycystic ovary syndrome (PCOS) have revealed aberrant profiles of natural killer (NK) cells. In the peripheral blood and spleen, NK cells exhibit altered subset frequency and receptor expression patterns. In the ovary and uterus, the activity and abundance of NK cells (including CD69+ cells and uterine natural killer (uNK) cells) are increased, with compromised functional capacity such as vascular remodeling. Hyperandrogenism and insulin resistance (IR) are the key drivers of these phenotypic and functional abnormalities in NK cells.

### 2.2 Human evidence

The frequency of circulating NK cells is generally higher in patients with PCOS than in healthy controls, which may be indicative of chronic inflammation. In the endometrium, the abundance of core uNK cells is decreased during the secretory phase, accompanied by cytokine dysregulation, whereas these cells display a distinct inflammatory profile in the proliferative phase. In addition, correlations have been identified between the frequency of specific killer immunoglobulin-like receptor (KIR) genes, NK cell abundance, and insulin sensitivity, obesity, as well as circulating inflammatory biomarkers.

### 2.3 Speculative translational hypotheses

Based on the aforementioned evidence, several hypotheses linking NK cell dysfunction to PCOS pathology have been proposed. First, elevated peripheral blood NK cell proportions may serve as a marker of chronic inflammation and could be used to predict PCOS-associated infertility. Second, impaired uNK cell function and dysregulated cytokine networks in the endometrium may disrupt the implantation microenvironment, potentially explaining reduced fertility and increased pregnancy loss risk. Third, hyperandrogenism may drive local inflammation in reproductive tissues by modulating NK cell activity (e.g., CD69 expression), leading to impaired folliculogenesis and endometrial dysfunction. Fourth, insulin resistance-induced myNK cells may exacerbate metabolic inflammation and glucose homeostasis disturbances through the IL-6/Stat3 pathway. Finally, KIR gene variants might affect endometrial receptivity and vascular remodeling by altering the balance of NK cell signaling. These hypotheses provide a novel rationale for developing immunomodulatory therapeutic strategies.

## 3 Phenotypic and functional alterations of NK cells with PCOS

### 3.1 Characteristics of peripheral blood NK cells with PCOS

Clinical studies reveal that the proportion of NK cells in peripheral blood lymphocytes is significantly elevated in infertile patients with PCOS compared to healthy controls (13.88%-22.28% vs. 8.82%- 17.00%) (8). Furthermore, the study also suggested that exceeding the threshold of 16.43% may represent a potential cutoff value, thereby reflecting the chronic low-grade inflammatory state and systemic immune dysregulation characteristics of this syndrome. However, ROC analysis of this cutoff revealed an error rate of approximately 30% for the model. Therefore, establishing a clinically validated threshold through larger cohort studies would be more conducive to predicting the inflammatory status in PCOS (8). However, investigations using DHEA-induced PCOS mice models and human PCOS samples have reported no significant alterations in peripheral NK cells, while studies in DHT-induced PCOS rat models even observed reduced peripheral NK cell levels (9–12). These discrepancies may arise from limited sample sizes, differences in animal strains, species-specific variations, distinct PCOS induction methods, or the use of different antibody markers for identifying NK cells.

**Figure 1:**
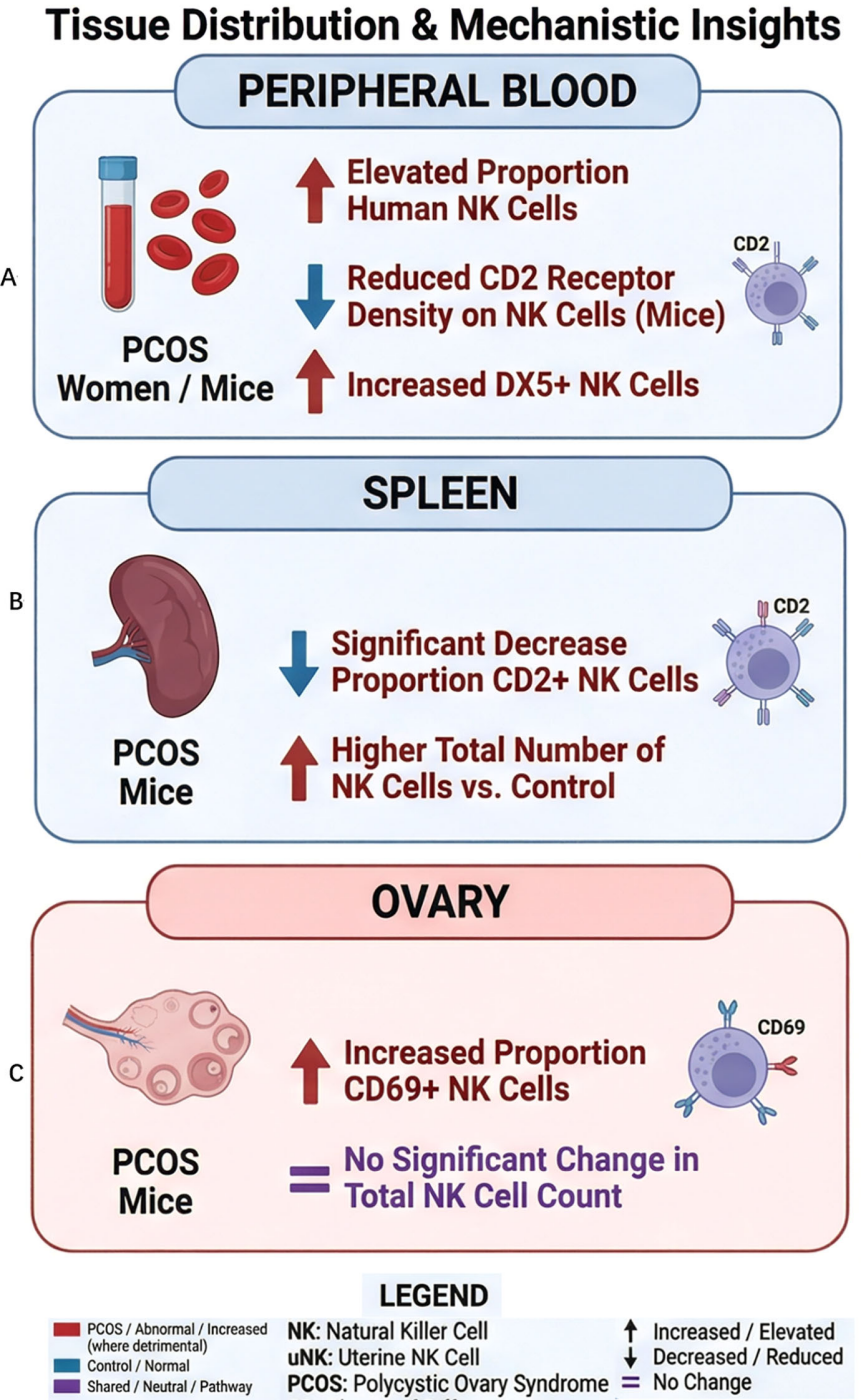
Tissue distribution and mechanistic insights of NK cells in PCOS. **(A)** Proportion of NK cells in peripheral blood of women with PCOS (8); CD2 receptor density and DX5 expression on NK cells in peripheral blood of PCOS mice (9, 10). **(B)** In the spleen of PCOS mice: altered proportion of CD2^+^ NK cells (9); comparison of total NK cell count (13). **(C)** In the ovary of PCOS mice: CD69^+^ NK cells; total NK cells (13). The legend defines the color codes and abbreviations used in the figure.

Mechanistic studies in obese PCOS mouse models reveal reduced surface density of the CD2 receptor on peripheral NK cells, while the count of CD2+ NK cells remains unchanged, indicating impaired expression of this activation-associated receptor (9). The increase in DX5+ NK cells further reflects an inflammatory state in these mice. In contrast, the expression of CD94 and CD69, as well as the numbers of CD94+ and CD69+ NK cells, show no significant alterations (9, 10, 13). The downregulation of CD2 may originate from chronic overstimulation by elevated serum IL-2. Such persistently high IL-2 levels could also diminish NK cell adhesion capacity and elevate their activation threshold (9, 14). Given that peripheral NK cells migrate via uterine arteries to the intervillous space and interact with syncytiotrophoblasts, reduced CD2 expression—a critical regulator of NK cell cytotoxicity—may weaken the maternal NK cell-trophoblast cross-talk. This impairment potentially contributes to the pathophysiology of infertility and elevated risk of neonatal complications observed in PCOS patients (15, 16) (**Figure 1**).

### 3.2 Characteristics of spleen NK cells in PCOS

Alterations in the phenotype, quantity, and activation status of NK cells within the spleen exhibit a close association with both immune dysregulation and endocrine disturbances in PCOS, potentially contributing to the pathophysiology of associated infertility. Similar to observations in peripheral blood, changes in splenic NK cell numbers in both animal and women with PCOS lack consensus. Animal model studies report inconsistent findings, demonstrating both increased and unaltered NK cell counts in the spleen, while investigations into human splenic NK cells remain relatively scarce (9, 12, 17).

In obese PCOS rat models, the total number of splenic NK cells remains largely unchanged; however, the proportion of CD2^+^ NK cells declines significantly. Mirroring observations in peripheral blood, neither the quantity nor the receptor density of CD94^+^ NK cells shows marked alteration (9). Metformin promotes the mobilization of NK cells from the spleen to the peripheral blood, yet it exhibits limited impact on rectifying the aberrant CD2 expression (9). Another study employing a DHT-induced PCOS mouse model reports a higher splenic NK cell count compared with controls, while the population of activated CD69^+^ NK cells remains comparable, however, anti-androgen treatment prevents the increase in splenic NK cells (13) (**Figure 1**).

### 3.3 Altered ovarian NK cells in PCOS

Direct evidence regarding the quantity, phenotype (such as receptor expression), and function of NK cells within the ovaries of women with PCOS remains scarce. Studies using DHEA-induced PCOS mouse models indicate an elevated proportion of activated CD69^+^ NK cells in ovarian tissue, while the total NK cell count shows no significant alteration—an effect reversible upon treatment with an androgen receptor antagonist (13). Although overall immune cell infiltration in the ovary is increased in PCOS animal models, a systematic quantification of ovarian NK cell numbers, detailed phenotypic characteristics (including activation receptor expression), and cytotoxic functions has yet to be delineated (18) (**Figure 1**).

## 4 Characteristics of endometrial NK cells in PCOS.

Uterine natural killer (uNK) cells represent the predominant innate immune population in the endometrium and are regarded as a key endometrial immunological hallmark of PCOS (19). Unlike their peripheral blood counterparts, which exhibit strong cytotoxicity, uNK cells (characterized by the CD56brightCD16− phenotype) possess relatively low cytotoxic activity. Instead, they primarily function as immunomodulators, secreting cytokines such as IFN-γ and TNF-α during the secretory phase of the endometrium (7). While IFN-γ plays a profound role in spiral artery remodeling, TNF-α is indispensable for successful embryo implantation, together maintaining normal endometrial function (20, 21). uNK cells occupy a central position in regulating local immune tolerance, inflammatory responses, and endometrial angiogenesis and vascular remodeling. Beyond defending the uterine mucosa against external challenges, these cells actively contribute to female reproductive homeostasis and participate in multiple physiological events, including follicular development, ovulation, and the menstrual cycle (22). In the setting of chronic endocrine and metabolic disturbances characteristic of PCOS, alterations in the abundance, phenotype, or function of uNK cells are likely to disrupt the endometrial microenvironment and compromise its receptivity (23). Endometrial immune dysregulation in PCOS, potentially linked to aberrant NK cell activities and cytokine network imbalances, may be further amplified by insufficient progesterone levels or hyperandrogenism, which collectively perturb endometrial immune equilibrium via effects on uNK cells (24).

### 4.1 Role of NK cells and their cytokine network in endometrial function

In the normal endometrium, the decidualization process guided by progesterone promotes an increase in the proportion of CD56bright NK cells. These cells contribute to an optimal immune microenvironment for embryo implantation through the release of regulatory molecules (25). Although progesterone does not act directly on NK cells, it upregulates CXCL10 expression, facilitating the homing and proliferation of peripheral NK cells (26, 27). Concurrently, it regulates the expression of IL-15 and IL-18 during endometrial decidualization, thereby modulating the proliferation of existing uterine natural killer (uNK) cells (28–31).

The secretory-phase endometrium of infertile women with PCOS exhibits a marked imbalance in immune cell populations. The proportion of immunoregulatory CD56+/CD16- NK cells, particularly uNK cells, is significantly reduced (11). This decline coincides with diminished local expression of key signaling molecules—IL-15, IL-18, and CXCL10 (11). The dysregulation of this cytokine network, likely originating from the progesterone deficiency common in PCOS, ultimately impairs the recruitment and function of uNK cells.

**Figure 2:**
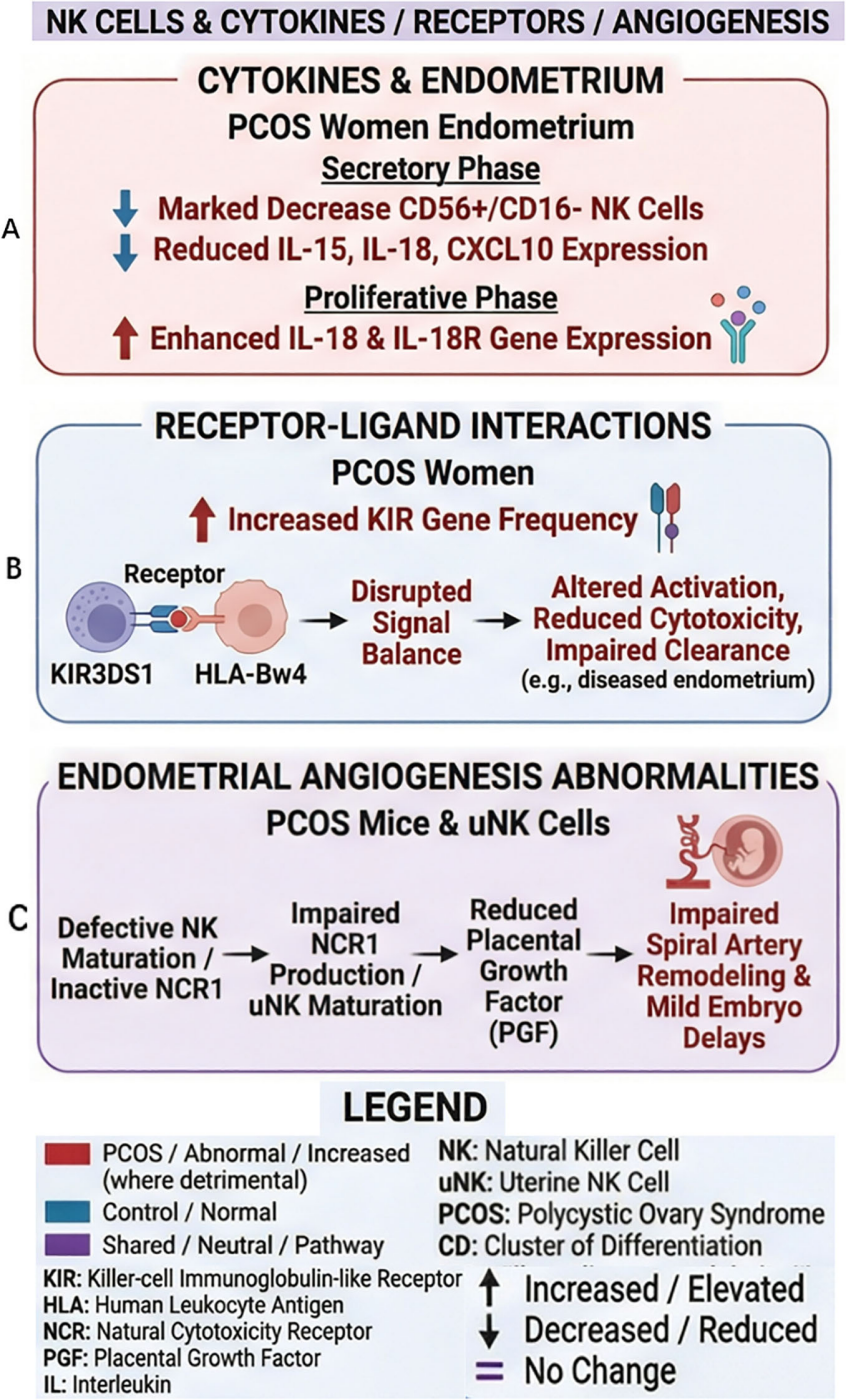
Interactions of NK cells in PCOS. **(A)** In the endometrium of patients with PCOS: secretory phase-altered numbers of CD56^+^/CD16^-^ NK cells, changes in cytokine expression (11). proliferative phase-altered gene expression of IL-18 and the IL-18 receptor (9). **(B)** Effect of increased KIR gene frequency in women with PCOS (5). **(C)** In PCOS mouse models: consequences of NCR1 deficiency in uNK cells (37). The legend defines the color codes and abbreviations used in the figure.

In contrast, the proliferative-phase endometrium in PCOS women presents a distinct pro-inflammatory profile. Studies on chronic endometrial inflammation in PCOS reveal elevated gene expression of the pro-inflammatory cytokine IL-18 and its receptor, IL-18R, in the proliferative endometrium. This upregulation is particularly pronounced in overweight women with PCOS, where increased endometrial IL-18 mRNA levels related to both the PCOS condition and body mass index, independently of IR (32, 33). This indicates that tissue-specific and temporal immune cell activation in PCOS may lead to stage-specific immune abnormalities. During the proliferative phase, this inflammatory background predisposes various endometrial immune cells to a “pre-activated” state, leading to the premature and excessive production of cytokines like IL-18. Subsequently, in the secretory phase, the impaired recruitment and functional maturation of uNK cells disrupts the establishment of endometrial receptivity. This cyclical immune dysfunction likely underlies the increased risks of implantation failure, early pregnancy loss, and even endometrial cancer observed in women with PCOS (**Figure 2**).

### 4.2 Endometrial function orchestrated by NK cells via surface receptor–ligand interactions

The functional status of NK cells hinges on the delicate equilibrium between signals from activating and inhibitory receptors, with killer immunoglobulin-like receptors (KIRs) playing a pivotal role. Under physiological conditions, the interaction between inhibitory KIRs (iKIRs) and their HLA ligands generates a dominant inhibitory signal that overrides the weaker activation signal from activating KIR (aKIR)-HLA engagement, thereby maintaining NK cells in a restrained state. A shift in this balance—particularly a reduction in inhibitory signaling—triggers NK cell activation, leading to cytotoxicity and cytokine production (34). The strength of these signals is influenced by KIR gene repertoire, individual KIR profiles, and the availability of specific HLA ligands.

Studies indicate an increased frequency of specific KIR genes in PCOS patients. For instance, the KIR3DS1-Bw4 combination occurs more frequently in affected individuals compared to healthy controls. This specific KIR3DS1 and HLA-Bw4 ligand interaction appears to disrupt signaling balance by promoting activating signal transduction, which suggests a decrease in NK cell cytotoxic function. However, whether the cytotoxic activity of NK cells is directly inhibited in PCOS patients still requires clarification through direct functional assays of NK cells. This impairment may reduce the clearance of aberrant cells within the endometrium, potentially exacerbating local inflammatory responses and compromising the endometrial microenvironment (5) (**Figure 2**).

### 4.3 Altered endometrial angiogenesis and NK cells

Dysfunctional NK cells, particularly those with compromised maturation or differentiation, can directly impair the endometrial capacity to generate angiogenesis-related factors. NCR1 (NKp46) is a highly conserved transmembrane receptor expressed across all developmental stages of activated NK cells in both mice and humans. In uNK cells, it fulfills unique functions, such as by promoting uNK cell maturation and influencing the expression of granule-associated proteins (e.g., perforin, granzymes) and cytoplasmic proteins (e.g., vascular endothelial growth factor, placental growth factor), thereby helping to impact pregnancy outcomes (35–37). Animal studies demonstrate that inactivation of the natural cytotoxicity receptor NCR1 disrupts uNK cell maturation and placental growth factor (PGF) production. In NCR1-deficient mouse models, this receptor loss leads to aberrant NK cell phenotypes, impaired spiral artery remodeling, and mild developmental delays in embryos, underscoring the essential role of intact NK cell function in maintaining vascular homeostasis (37) (**Figure 2**).

## 5 Role of NK cells in endocrine disturbances of PCOS

### 5.1 Hyperandrogenism with NK cells

Hyperandrogenemia in PCOS is closely linked to functional and distributional abnormalities in NK cells. Androgens may promote immune dysregulation and chronic inflammation in PCOS by altering NK cell distribution, phenotype, and activity. This pathway could represent one mechanism underlying the reproductive dysfunction associated with the syndrome, as supported by data from animal and clinical studies.

Androgen excess participates in the pathogenesis of chronic low-grade inflammation and reproductive impairment in PCOS by altering NK cell phenotypic and functional dynamics (38). In DHEA-induced PCOS mouse models, for instance, androgen exposure influences the expression of molecules such as CD2 and CD94, disrupting receptor-ligand interactions and impairing NK cell-mediated defense in the reproductive tract, folliculogenesis, and ovulation (13). Such aberrant activation and accumulation of NK cells may provoke localized inflammation within the uterus and ovary, potentially compromising follicular development and ovulation, which could be a contributing factor to infertility.

**Figure 3:**
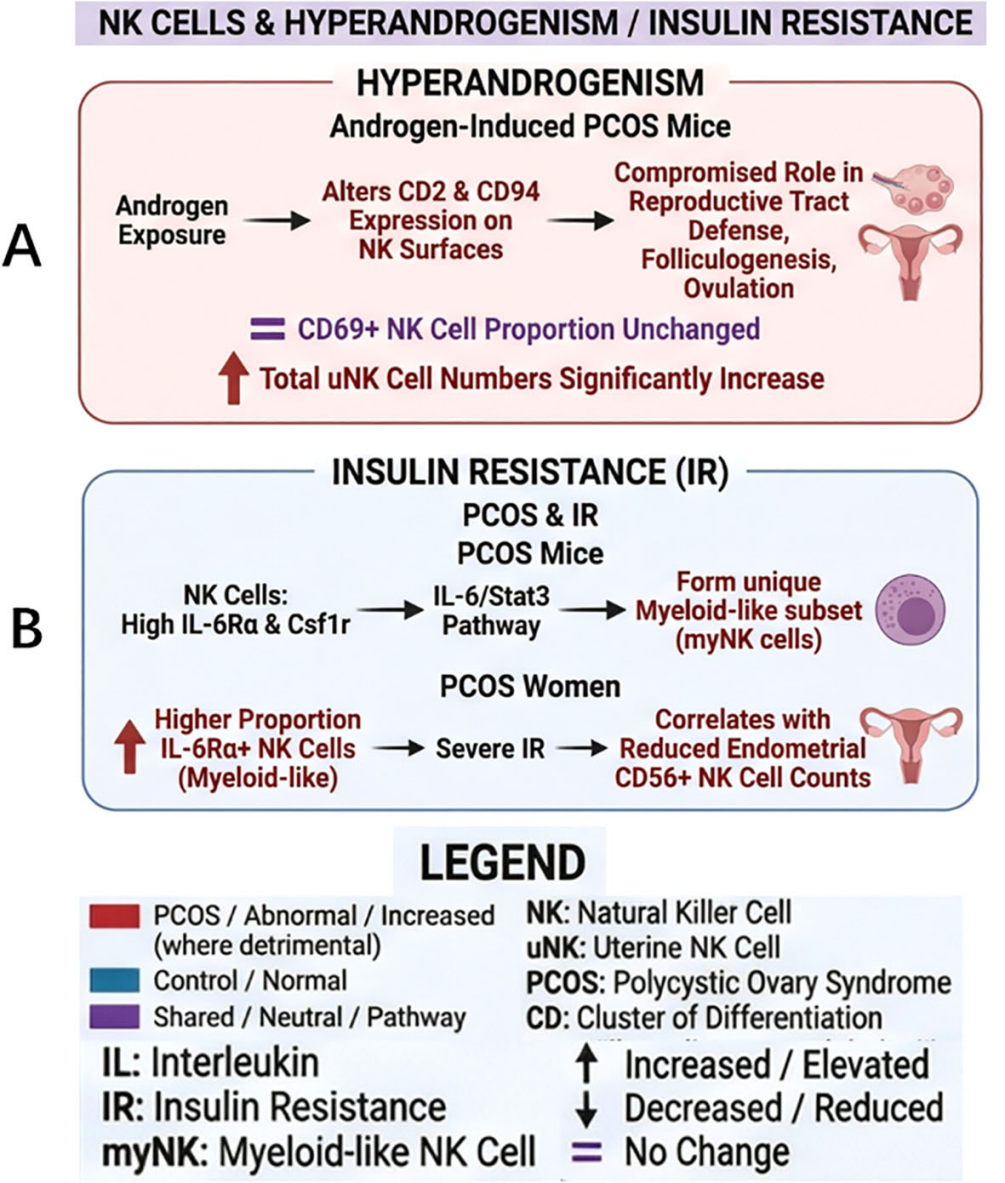
NK cells and endocrine disorders in PCOS. **(A)** In androgen-induced PCOS mouse models: alterations in NK cells and their associated effect (13). **(B)** Generation of myNK cell subsets in PCOS mouse models; effects of myNK-like cell subsets in PCOS women (33, 44). The legend defines the color codes and abbreviations used in the figure.

Further investigation into the mechanisms reveals that androgens also contribute to endometrial dysfunction. In DHEA-induced PCOS mouse models, uNK cells mirror observations in peripheral immune organs: while the proportion of NK cells expressing the activation marker CD69 remains stable, the total uNK cell population expands significantly. This effect can be reversed by an androgen receptor antagonist (13). These findings suggest that high androgen levels may directly alter the endometrial immune microenvironment, potentially impairing normal endometrial function by promoting excessive uNK cell proliferation and elevating pro-inflammatory cytokine levels (**Figure 3**).

### 5.2 Insulin resistance with NK cells

Insulin resistance is defined as a metabolic state characterized by a diminished biological responsiveness to physiological levels of insulin. Its core feature is a decreased sensitivity of insulin-target tissues—primarily skeletal muscle, liver, and adipose tissue—to insulin-mediated glucose uptake and utilization. There may be an association between insulin resistance and NK cell dysfunction in PCOS, and this connection could be underpinned by the specific inflammatory state characteristic (39–42). Levels of inflammatory mediators—such as C-reactive protein, TNF-α, and IL-6—are elevated in these patients, creating a self-sustaining vicious cycle in which inflammation and IR mutually reinforce each other.

Exosome miR-207 derived from NK cells of healthy mice carry microRNAs such as miR-1249-3p, which targets the SKOR1 protein. This interaction disrupts the assembly of the SMAD6/MYD88/SMURF1 complex, leading to suppression of the TLR4/NF-κB inflammatory signaling pathway. Consequently, this exosome-mediated mechanism alleviates obesity-induced IR and inflammation, demonstrating that NK cells can modulate metabolic homeostasis via paracrine signaling (43).

Studies using obese mouse models have revealed that NK cells in adipose tissue and peripheral blood aberrantly express receptors typically associated with myeloid cells, including interleukin-6 receptor (IL-6Rα) and colony-stimulating factor 1 receptor (Csf1r). These Csf1r−bearing NK cells constitute a distinct subset termed myeloid-feature NK (myNK) cells. Further investigation implicates the IL-6/Stat3 signaling pathway within NK cells as a key driver of myNK cell generation and metabolic dysfunction. Under obese conditions, persistently elevated IL-6 activates Stat3, promoting the expansion of myNK cells and exacerbating systemic metabolic inflammation and IR. In animal models, selective depletion of Csf1r^+^ (i.e., IL−6Rα^+^) myNK cells attenuates weight gain, reduces fat mass, and improves insulin sensitivity and glucose tolerance, along with lowering circulating proinflammatory cytokine levels (44).

This mechanism finds parallels in human studies. Obese individuals exhibited significantly higher proportions and expression levels of IL-6Rα^+^ myNK cells in the peripheral circulation compared to those with normal body weight. The number of these cells showed a positive correlation with the level of high-sensitivity C-reactive protein (hsCRP) in obese subjects, a marker of systemic low-grade inflammation. This correlation suggests that IL-6Rα^+^ myNK cells may likewise play a pro-inflammatory role and drive metabolic disturbances in humans. Gene expression profiling of these human IL−6Rα^+^ NK cells also reveals a similar myeloid−like gene signature. The concordance of NK cell phenotypic alterations and IR improvement in both animal and human studies underscores the direct role of NK cells in metabolic regulation (44).

Finally, within the endometrium of PCOS patients, the number of CD56^+^ NK cells showed a positive correlation with insulin sensitivity, suggesting that reduced endometrial CD56^+^ NK cell counts are associated with IR in women with PCOS. This phenomenon appears to be independent of body weight, as no significant differences were observed between normal−weight and overweight PCOS subjects, or between overweight and normal−weight control groups, implying that the link may be intrinsic to PCOS pathology (33) (**Figure 3**).

## 6 Impact of NK cell phenotypic heterogeneity on research

Heterogeneity in NK cell research outcomes likely stems from their intrinsic high degree of phenotypic diversity and methodological variations across studies. NK cells comprise functionally distinct subsets (e.g., cytotoxic subsets in peripheral blood versus immunoregulatory uterine NK [uNK] cells in the endometrium). The use of different identification markers (e.g., CD56, DX5, CD2) and diverse animal models (e.g., DHEA-induced, DHT-induced) leads to a focus on non-identical phenotypes, rendering the resulting data difficult to compare directly.

This inconsistency significantly undermines both the reproducibility of research and its clinical translation. Discrepancies in experimental protocols hinder the replication of findings across laboratories, posing a major challenge to establishing stable and reliable diagnostic biomarkers (such as thresholds for peripheral blood NK cell proportions). Furthermore, uncertainty persists regarding whether specific phenotypic alterations observed in animal models (e.g., myNK cells) accurately reflect human pathology. This ambiguity complicates the transition from mechanistic insights to the development of precise immunomodulatory therapies.

## 7 Summary and outlook

Women with polycystic ovary syndrome (PCOS) exhibit marked natural killer (NK) cell dysfunction, an immune imbalance that permeates the entire spectrum of reproductive and metabolic disturbances characteristic of the disorder. While some studies suggest an elevated proportion of peripheral blood NK cells in humans, other research does not support this view. Furthermore, in the endometrium, uterine natural killer (uNK) cells—which are crucial for immune regulation—have been shown to exhibit reduced numbers and impaired function. This local deficit coincides with dysregulation of cytokine networks involving IL-15 and IL-18, severely compromising the microenvironment essential for embryo implantation. Altered expression profiles of NK cell surface receptors, such as killer immunoglobulin-like receptors (KIR), further contribute to diminished endometrial receptivity and aberrant vascular remodeling. Mechanistically, hyperandrogenemia modulates the distribution and activity of NK cells in reproductive tissues by regulating activation markers like CD69, while IR promotes the emergence of a myNK cell subset via the IL-6/Stat3 signaling pathway. Both ways could contribute to worsened chronic inflammation and metabolic dysregulation in PCOS. Current research faces several challenges, including inconsistencies across different experimental models, a lack of direct evidence characterizing NK cells within the ovarian tissue, and insufficient proof for a direct link between NK cells and insulin resistance (IR) in PCOS. Future studies will require larger-scale human trials combined with more direct animal models and NK cell-based assays to elucidate whether and how changes occur in the quantity, distribution, phenotype, and function of NK cells across various tissues in patients with PCOS, thereby informing novel immunomodulatory therapeutic strategies.
